# Altered lung tissue lipidomic profile in caspase-4 positive non-small cell lung cancer (NSCLC) patients

**DOI:** 10.18632/oncotarget.27724

**Published:** 2020-09-22

**Authors:** Michela Terlizzi, Antonio Molino, Chiara Colarusso, Pasquale Somma, Ilaria De Rosa, Jacopo Troisi, Giovanni Scala, Rosario Salvi, Aldo Pinto, Rosalinda Sorrentino

**Affiliations:** ^1^Department of Pharmacy, DIFARMA, University of Salerno, Fisciano, Salerno, Italy; ^2^ImmunePharma S.r.l., Salerno, Italy; ^3^Department of Clinical and Surgical Medicine, University of Naples Federico II, Naples, Italy; ^4^Anatomy and Pathology Unit, Ospedale dei Colli, AORN, “Monaldi”, Naples, Italy; ^5^Hosmotic Srl, Vico Equense, Naples, Italy; ^6^Theoreo Srl, Pugliano, Salerno, Italy; ^7^Department of Medicine, Surgery and Dentistry, “Scuola Medica Salernitana”, University of Salerno, Baronissi, Salerno, Italy; ^8^European Biomedical Research Institute of Salerno (EBRIS), Salerno, Italy; ^9^Thoracic Surgery Unit, Ospedale dei Colli, AORN, “Monaldi”, Naples, Italy; ^*^These authors contributed equally to this work

**Keywords:** NSCLC, metabolomic, lipidomic, metabotype, caspase-4

## Abstract

Lung cancer is by far the leading cause of cancer death. Metabolomic studies have highlighted that both tumor progression and limited curative treatment options are partly due to dysregulated glucose metabolism and its associated signaling pathways. In our previous studies, we identified caspase-4 as a novel diagnostic tool for non-small cell lung cancer (NSCLC). Here, we analyzed the metabolomic profile of both plasma and tumor tissues of NSCLC patients stratified as caspase-4 positive or negative. We found that circulating caspase-4 was correlated to LDH. However, this effect was not observed in caspase-4 positive tumor tissues, where instead, fatty acid biosynthesis was favoured in that the malonic acid and the palmitic acid were higher than in non-cancerous and caspase-4 negative tissues. The glycolytic pathway in caspase-4 positive NSCLC tissues was bypassed by the malonic acid-dependent lipogenesis. On the other hand, the dysregulated glucose metabolism was regulated by a higher presence of succinate dehydrogenase (SDHA) and by the gluconeogenic valine which favoured Krebs’ cycle.

In conclusion, we found that the recently identified caspase-4 positive subpopulation of NSCLC patients is characterized by a lipidomic profile accompanied by alternative pathways to guarantee glucose metabolism in favour of tumor cell proliferation.

## INTRODUCTION

Lung cancer still represents the leading cause of cancer-related mortality worldwide [1]. However, when cancer is detected at earlier stages, survival rate ameliorates. So far, several screenings have been developed to identify biomarkers to provide insights in diagnosis. In particular, genomic and proteomic data have provided advances to early detect the disease [2, 3]. The metabolomic area is complementary to genomics and proteomics in that it can provide further information about lung tumor formation and progression. Alteration of tumor cell metabolism is a concept that has been identified as one of the hallmarks of carcinogenesis [4]. Tumor cells have encompassed the capability to acquire nutrients in a different manner than healthy cells in order to provide long-ranging cell-fate and guarantee tumorigenesis. In this context, changes in tumor cells metabotype consist in deregulated metabolism of glucose and proteins, opportunistic modes of nutrient acquisition, use of glycolysis and tricarboxylic acid (TCA) cycle, increased regulation of nucleotide and polyamine biosynthesis, alterations in metabolite-driven gene regulation, metabolic interaction with the microenvironment [5]. So far, the literature identifies metabolites that are common to both cancerous tissues and serum which may serve not only as biomarkers but also as indicators of disease progression (i.e., lactate dehydrogenase (LDH) [6]. Moreover, besides derivatives from glucose metabolism, serum of lung cancer patients shows decreased circulating fatty acids and lipids in that they are required for growth, proliferation, invasion and metastasis in the tumor environment [7].

In our previous study we found circulating and tumor-associated caspase-4 as a novel diagnostic, predictive and prognostic biomarker for non-small cell lung cancer (NSCLC) patients [8]. Therefore, according to the altered metabotype of lung cancer patients, the main goal of the actual study was to understand any correlation between caspase-4 and the metabolomic profile of NSCLC patients by using both lung tumor tissues and plasma. We found that caspase-4 positive NSCLC patients had a differential lipidomic signature in that both the palmitic and the malonic acid were higher present in the tumor mass of caspase-4 positive rather than negative NSCLC patients.

## RESULTS

### High levels of plasma lactate dehydrogenase (LDH) are associated to caspase-4 positivity in NSCLC patients

**Figure 1:**
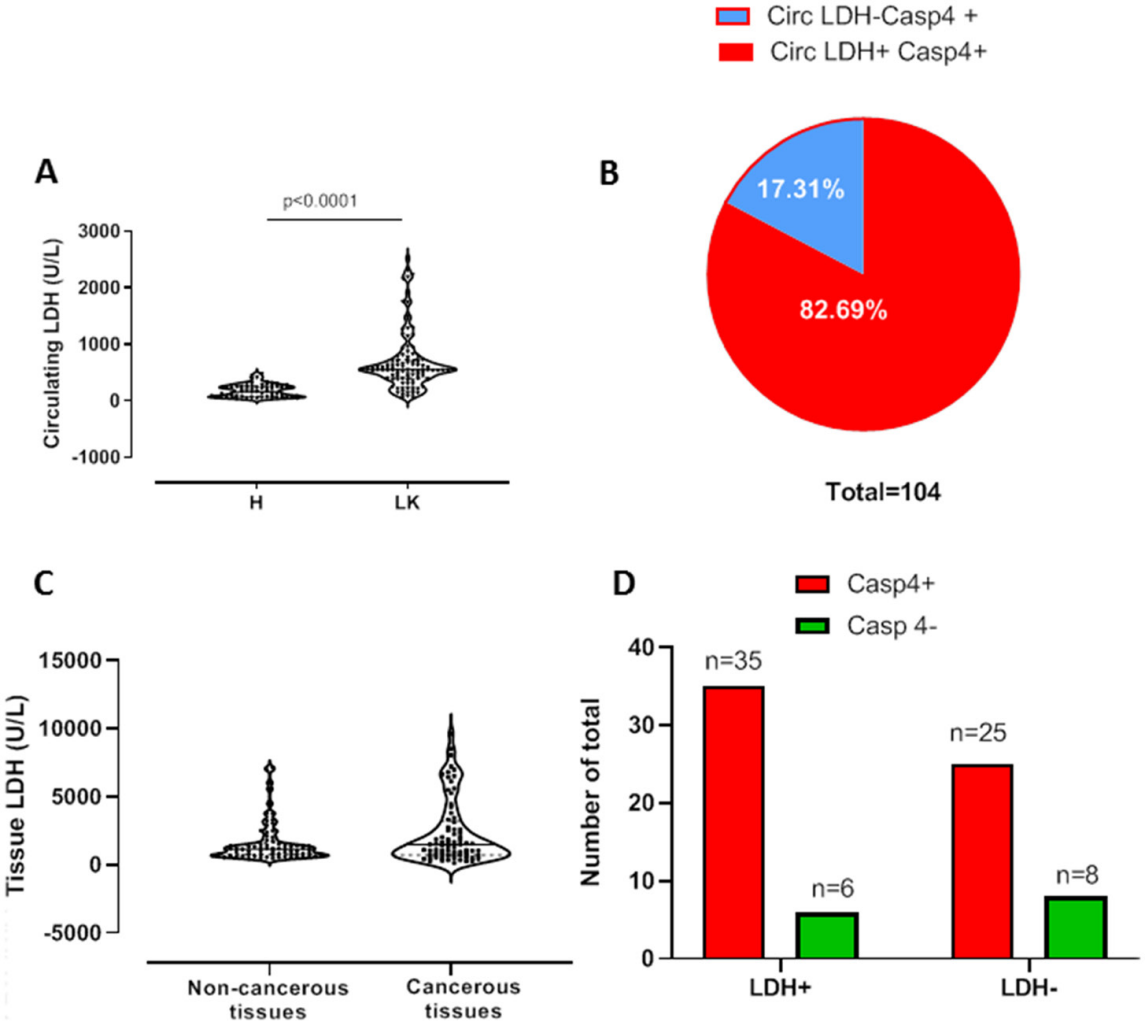
High levels of plasma, but not of tissue, LDH are associated to caspase-4-positive NSCLC patients. (**A**) Plasma/circulating levels of LDH were evaluated in healthy (H, *n* = 61) and NSCLC (LK, *n* = 104). (**B**) Patients who were positive (red part, 82,69%) or negative (blue part, 17,31%) to plasma LDH were represented as fraction of total caspase-4 positive patients. (**C**) Tissue levels of LDH in matched pairs of non-cancerous and cancerous tissues obtained by NSCLC patients undergoing thoracic surgery. (**D**) Representation of caspase-4 positive (red bars) and caspase-4 negative (green bars) according to a cut-off value of LDH levels, identified by ROC analysis (1182 U/L). Data are showed as median ± interquartile range and represented as violin plots. Two-tailed Mann Whitney *U* test was applied. *p* < 0.05 was considered as significant.

High levels of LDH are widely associated to chronic inflammatory diseases as well as to tumor progression/prognosis [9, 10]. In our previous study we found that caspase-4 is a novel diagnostic tool for NSCLC patients [8]. Therefore, we first evaluated the levels of circulating (plasma) LDH in NSCLC patients compared to healthy subjects and then correlated them with circulating caspase-4. As expected, NSCLC patients had significantly higher plasma levels of LDH than healthy subjects (Figure 1A). In order to correlate the levels of plasma LDH to caspase-4, we used a clinical practice cut-off for LDH as 240 U/L. We found that 82.69% of caspase-4 positive patients (86 out of 104) had plasma levels of LDH higher than 240 U/L (Figure 1B, red part). Instead, 17.31% of caspase-4 positive NSCLC patients (18 out of 104) did not have levels of LDH higher than 240 U/L, herein identified as LDH negative (LDH-) (Figure 1B, blue part).

Because LDH is a key enzyme in the conversion of the pyruvate to lactate under anaerobic conditions and links to tumor progression and prognosis [6, 9, 10], we went on by evaluating tissue-derived LDH and correlated it to the levels of tissue caspase-4. We used matched–pairs of non-cancerous and cancerous lung tissues obtained by patients undergoing surgical resection of the tumor mass. The levels of LDH in non-cancerous tissues did not statistically differ from those observed from cancerous tissues (Figure 1C). However, because plasma data indicated that 82.69% of caspase-4 positive patients had higher levels of LDH, we wanted to correlate the two markers in the tissues. To do so, according to ROC analysis, we defined a cut-off value for LDH in the tissue that was of 1182 U/L, value that, in our experimental conditions, corresponded to the value at which sensitivity and specificity were identical around 52%. We found that 35 patients out of 74 (Figure 1D, red bar on the left), representing 47.3% of our database (Table 1), were positive to tissue LDH and tumor-associated caspase-4. Instead, 6 out of 74 (Figure 1D, green bar on the left), representing 8.11% of NSCLC patients (Table 1), were positive to tissue LDH but not to caspase-4. Moreover, 33.78% of tissue-caspase-4 positive NSCLC patients (25 out of 74) were LDH negative (Figure 1D, red bar on the right) (Table 1), implying that in the tumor mass, differently than what observed in the blood, a differential metabolic pattern can exist.

**Table 1:** NSCLC patients positive to caspase-4 and LDH

	Casp 4+	Casp 4–
Tissue LDH+	35 (47.3%)	6 (8.11%)
Tissue LDH–	25 (33.78%)	8 (10.81%)

### Metabolomic signature in caspase-4 positive NSCLC patients

**Figure 2:**
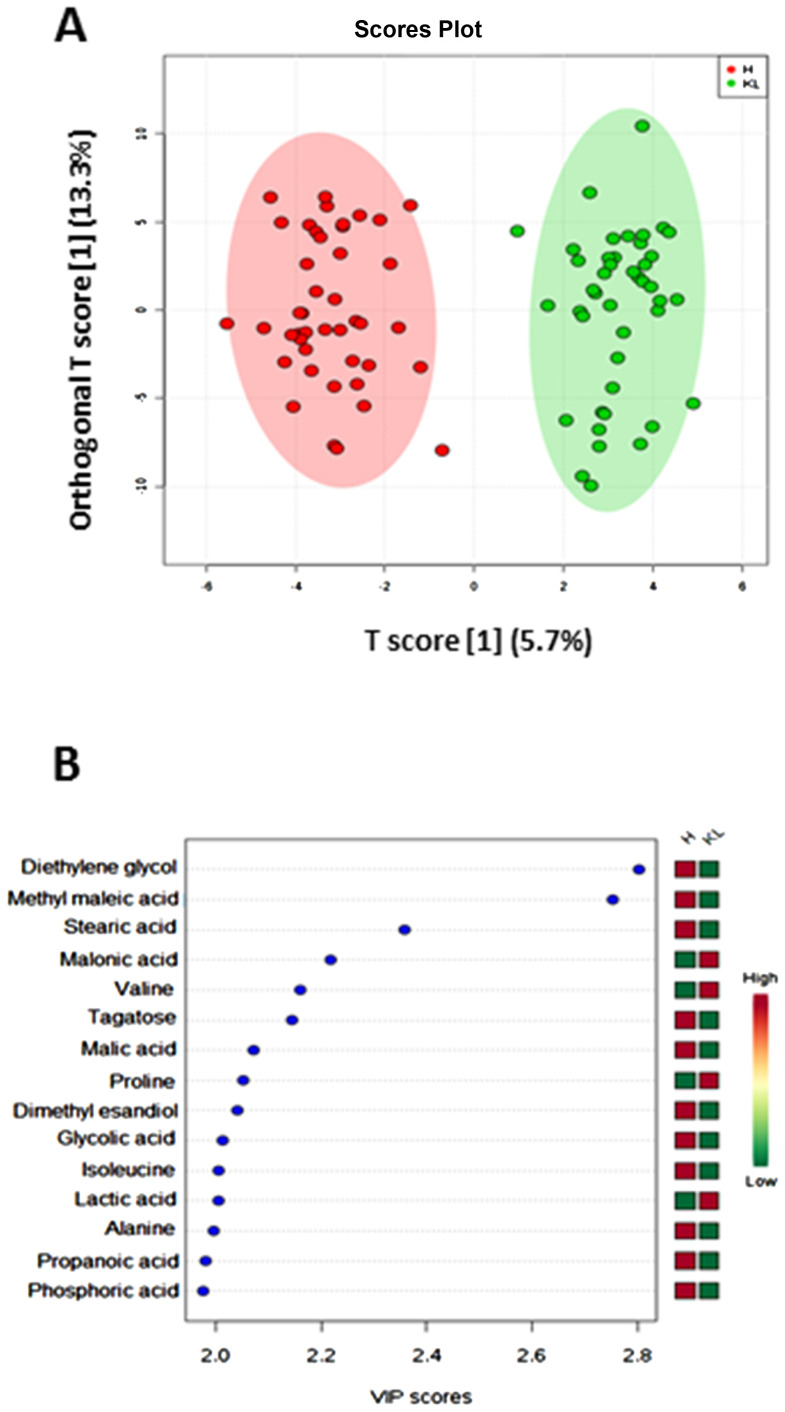
Metabolomic profile of NSCLC patients. Partial least squares-discriminant analysis (PLS-DA) of tissue-derived metabolites determined by means of GC-MS. (**A**) Two dimensional score plot showing clustering and separation between non-cancerous (healthy, H, green symbols) and cancerous (KL, red symbols) tissues. (**B**) Heatmap plot of the most relevant metabolites (VIP-score > 2.0).

**Figure 3:**
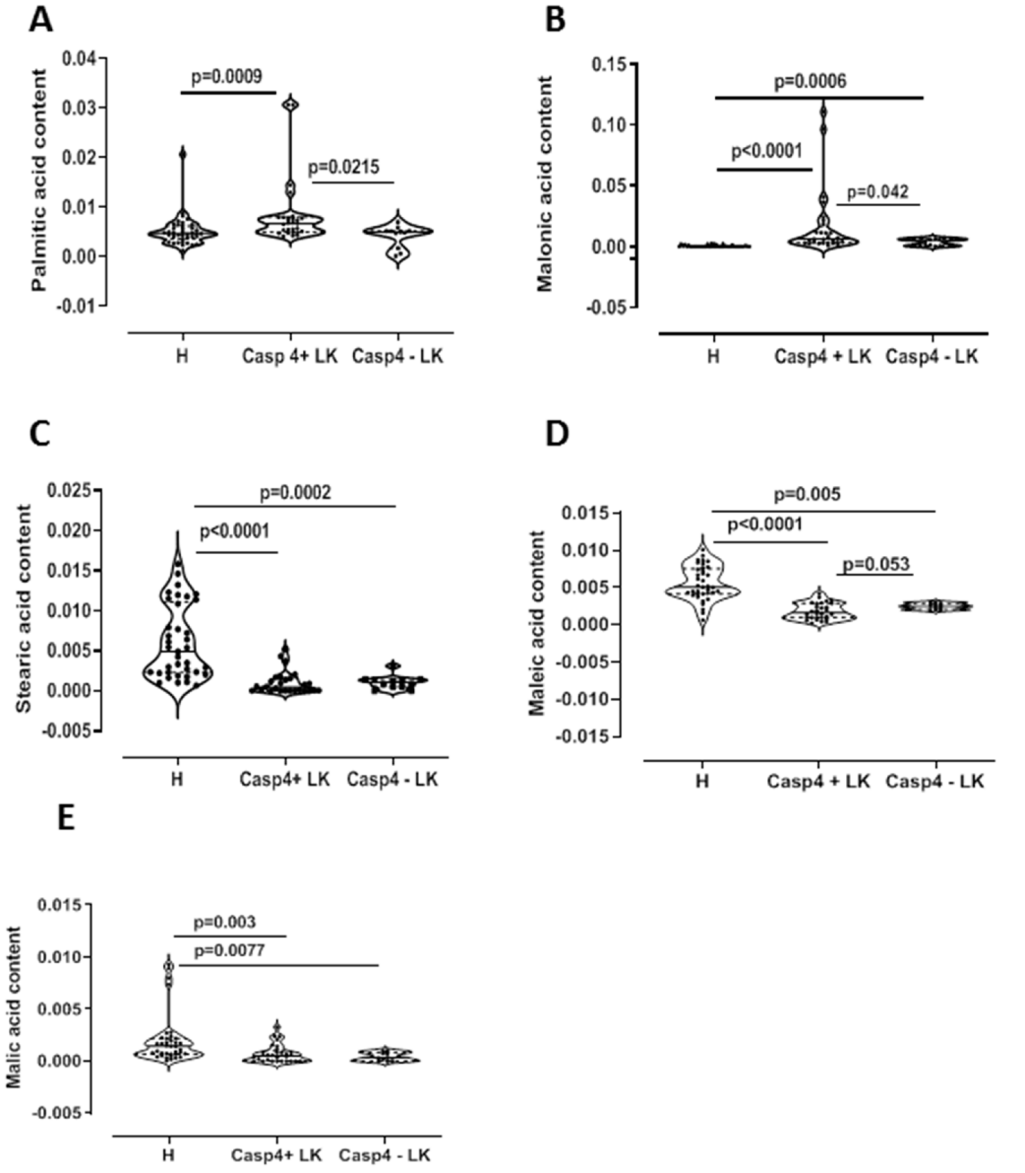
Lipidomic profile of NSCLC patients. GC-MS content of palmitic acid (**A**), malonic acid (**B**), stearic acid (**C**), maleic acid (**D**) and malic acid (**E**) in tissue caspase-4 positive (Casp4+ LK) or negative (Casp4 – LK) cancerous tissues vs non-cancerous (H) tissues (*n* = 39). Data are showed as median ± interquartile range and represented as violin plots. One-Way ANOVA followed by Dunn’s multiple comparison post-test was performed. *p* < 0.05 was considered as significant.

**Figure 4:**
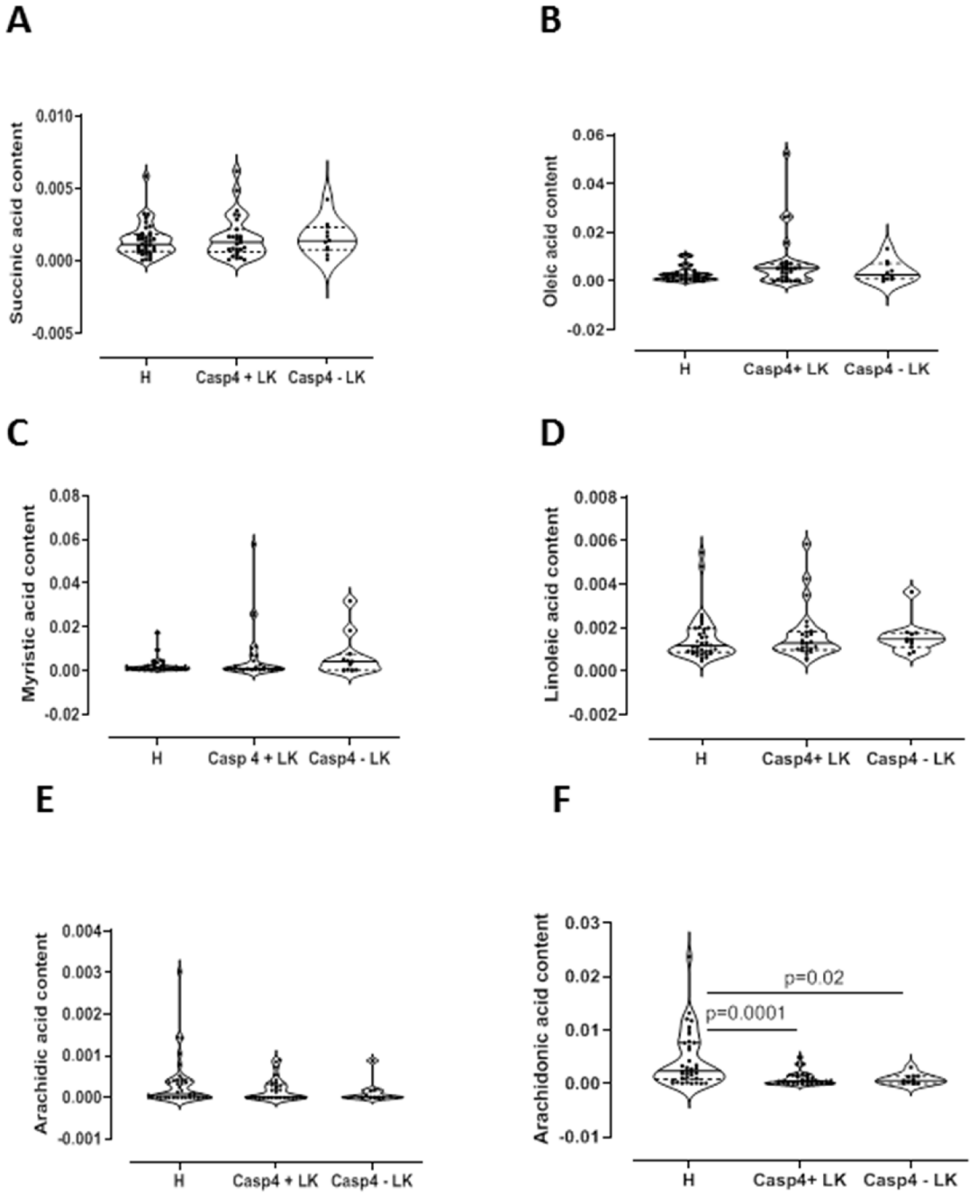
Metabolomic profile of NSCLC patients. GC-MS content of succinic acid (**A**), oleic acid (**B**), myristic acid (**C**), linoleic acid (**D**), arachidinc acid (**E**) and arachidonic acid (**F**) in tissue caspase-4 positive (Casp4+ LK) or negative (Casp4 – LK) cancerous tissues vs non-cancerous (H) tissues (*n* = 39). Data are showed as median ± interquartile range and represented as violin plots. One-Way ANOVA followed by Dunn’s multiple comparison post-test was performed. *p* < 0.05 was considered as significant.

In order to understand tissue metabotype in caspase-4 positive NSCLC patients, we performed an untargeted metabolomic analysis. Matched-pair non-cancerous (reported as H, that is healthy) and cancerous tissue (reported as LC, that is lung cancer) homogenates showed two-dimensional score plots discriminating two clusters (Figure 2A), and identifying specific relevant metabolites as highly present in cancerous compared to non-cancerous tissues (Figure 2B). In particular, lactic acid, proline, valine and malonic acid had very high variable importance in projection (VIP) scores in cancerous than non-cancerous tissues (Figure 2B). We moved on by evaluating the levels of these metabolites according to the levels of caspase-4 in the tumor mass based on a previous identified cut-off (0.307 ng/ml) [8]. Tumor-positive caspase-4 tissues had significantly higher levels of palmitic acid than both healthy (non-cancerous) and tumor-negative caspase-4 tissues (Figure 3A). Similarly, we found that malonic acid content was significantly higher in tumor-positive caspase-4 tissues than in healthy, non-cancerous, and tumor-negative caspase-4 tissues (Figure 3B). To note, both palmitic acid and malonic acid contents were higher in tumor-positive than tumor-negative caspase-4 tissues (Figure 3A and 3B). Instead, a significant reduction of stearic acid (Figure 3C), maleic acid (Figure 3D) and malic acid (Figure 3E) was observed in both tumor-positive and negative caspase-4 tissues compared to healthy, non-cancerous tissues. Moreover, no statistical differences were observed among the tissues (healthy, non-cancerous, vs tumor-caspase-4 + and caspase-4 -) about the contents of succinic acid (Figure 4A), oleic acid (Figure 4B), myristic acid (Figure 4C), linoleic acid (Figure 4D) and arachidic acid (Figure 4E), but not of arachidonic acid (Figure 4F). In this latter case, a significant reduction of the arachidonic acid was observed in both tumor-positive and negative caspase-4 tissues, implying the involvement of leukotrienes, lipoxins and prostaglandins [11] in the tumor tissue.

To note, the same analysis was performed in the plasma of NSCLC patients and compared to the plasma of healthy subjects (Supplementary Figure 1). No differences were noted in the metabolomic content, unless for palmitic acid (Supplementary Figure 1A), stearic acid (Supplementary Figure 1C), malic acid (Supplementary Figure 1E), arachidic acid (Supplementary Figure 1F) and myristic acid (Supplementary Figure 1H). In particular, the palmitic acid had opposite tendency in the plasma than in the tissue where levels were higher (Figure 3A).

### Proteomic signature in caspase-4 positive NSCLC patients

**Figure 5:**
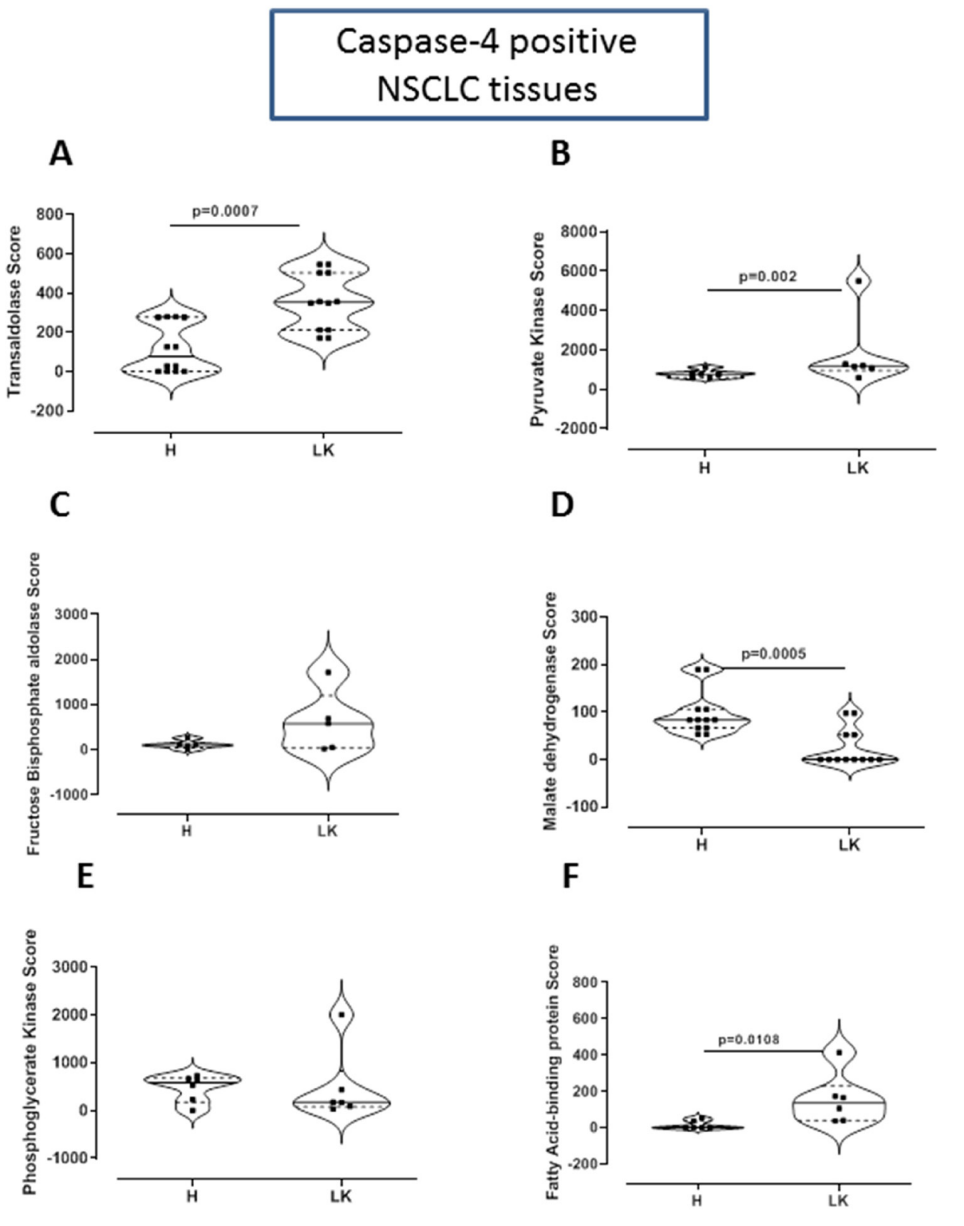
Proteomic profile of caspase-4 positive NSCLC tissues. Proteomic data was analyzed by MASCOTT software following LC-MS of trypsin-digested proteic bands obtained after SDS-page of collagenase-digested lung tissues of non-cancerous (H) and cancerous (LK) tissues. Tissue proteic levels of (**A**) transaldolase, (**B**) pyruvate kinase, (**C**) fructose bisphoshate aldolase, (**D**) malate dehydrogenase, (**E**) phosphoglycerate kinase and (**F**) fatty acid binding protein. Samples are match-paired and were obtained from NSCLC patients undergoing thoracic surgery. Data are showed as median ± interquartile range and represented as violin plots. Two-tailed Mann Whitney *U* test was performed. *p* < 0.05 was considered as significant.

Because the above data was referred to a metabolomic profile, we went on by analyzing the proteomic profile in caspase-4 positive NSCLC patients to evaluate enzymes involved in metabolic pathways. Caspase-4 positive tumor tissues had significantly higher levels of transaldolase (Figure 5A) and pyruvate kinase (Figure 5B). The levels of fructose bisphosphate aldolase were likely higher in tumor tissues, although they did not reach statistical significance (Figure 5C). Instead, malate dehydrogenase (Figure 5D) and phosphoglycerate kinase (Figure 5E) tended to decrease in tumor tissues, implying that higher consumption of glucose occurred. In support to our previous data on the lipidomic profile, higher levels of fatty acid-binding protein were found in caspase-4-positive tumor tissues (Figure 5F).

**Figure 6:**
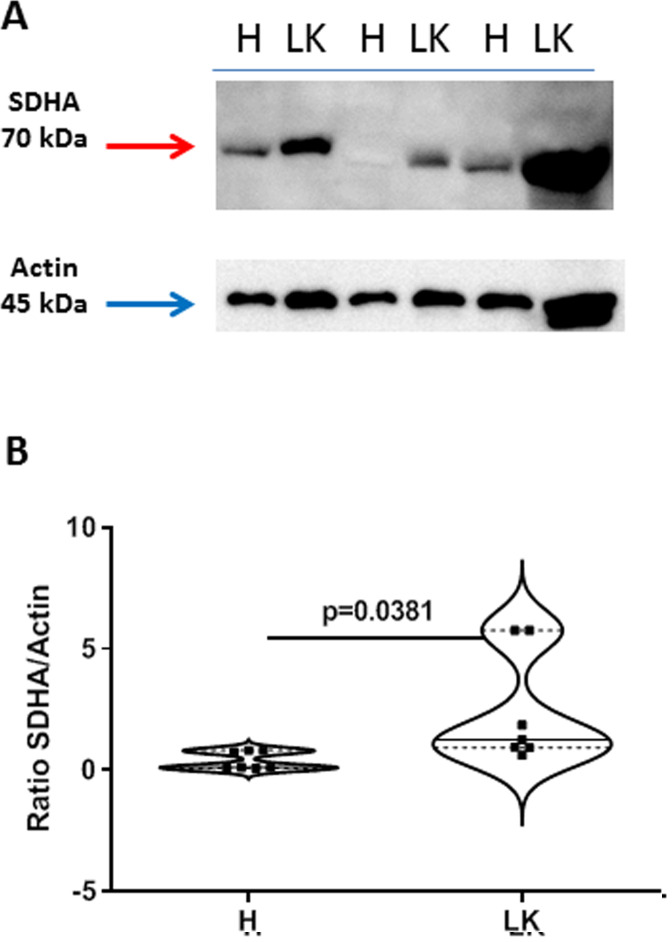
Expression of succinate dehydrogenase (SDHA) in non-cancerous (H) and cancerous (LK) collagenase-digested NSCLC tissues. (**A**) Representative western blots are shown. (**B**) Data are expressed as ratio SDHA (70 kDa) vs actin (45 kDa), used as loading control. Data are showed as median ± interquartile range and represented as violin plots. Two-tailed Mann Whitney *U* test was performed. *p* < 0.05 was considered as significant.

Because the above enzymes are involved in the glycolytic pattern, it appeared that the tumor tissue was not able to provide energy to the cell. Instead, we found that succinate dehydrogenase (SDHA) was higher expressed in the tumor mass of caspase-4 positive NSCLC patients compared to the non-cancerous tissues (Figure 6A and 6B).

## DISCUSSION

Metabolic alterations are associated to pathological conditions. It is widely reported that the alteration of the metabolomic profile in cancer represents one of the hallmarks for tumor cells which hijack the physiological pathways opportunistically. In this context, we found that caspase-4-positive NSCLC patients presented a metabolomic shift towards a lipidomic profile in that the palmitic and the malonic acid were higher present.

**Figure 7:**
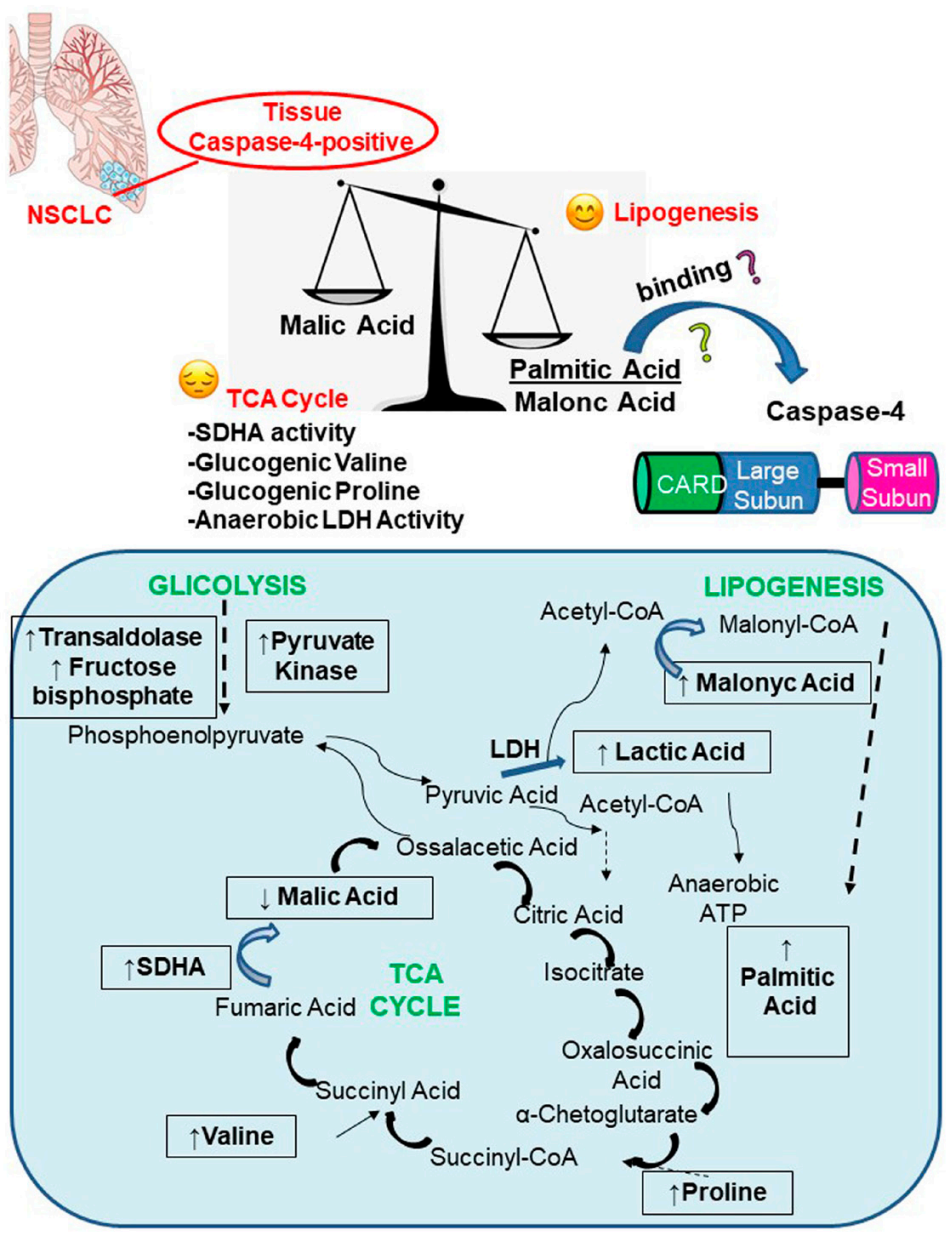
Caspase-4 positive NSCLC tissues present a pronounced lipogenic profile due to palmitic and malonic acid formation despite the TCA cycle, which seemed to be unbalanced. Herein, the equilibrium is reached via the higher presence of SDHA, Valine, Proline which tend to recover the unbalanced-TCA cycle to provide ATP to tumor cells. Boxes in the schematic represent the metabolites that have been detected in caspase-4 positive tumor tissues compared to non-cancerous tissues.

Alterations in serum levels of fatty acids and lipids between healthy subjects and NSCLC patients have been already reported [12, 13]. Reduced plasma lipid contents were associated to lung cancer progression in that they favor tumor cell proliferation and progression/metastasis. In our study, we similarly found that lipid contents in NSCLC patients were reduced in the blood, but instead, in the tissue we found that higher levels of lipid contents were observed in caspase-4-positive NSCLC patients. In our previous study, we identified both circulating and tissue-associated caspase-4 as a novel diagnostic tool for NSCLC patients. In our cohort, NSCLC patients were all positive to circulating levels of caspase-4 [8]. Among these patients, solely 82.7% of plasma caspase-4 positive NSCLC patients were positive to plasma LDH according to the clinical cut-off. LDH has been often associated to inflammasome-dependent pyroptosis [14, 15] in that the activation of inflammatory caspases (i.e., caspase-1 and caspase-11 or caspase-4) leads to the release of alarmins that induce cells to death identified by the released extracellular LDH. In the same manner, LDH is widely recognized as a marker associated to tumor progression and prognosis, which on one side could represent tumor cell necroptosis or on the other the anaerobic pattern that is established in the tumor mass [6, 9, 10]. In our experimental conditions, tissue levels of both caspase-4 and LDH dissected NSCLC patients in a smaller subpopulation (47.3%), implying on one side that LDH and caspase-4 are not strictly correlated at the tissue levels, but on the other that caspase-4 is not able to induce cell death in the tumor tissue. In particular, these data suggested that besides the anaerobic production of ATP (Figure 7), additional pathways influenced the metabotype of caspase-4 positive NSCLC patients. Indeed, we found that both the palmitic and the malonic acid were increased in the tissue of caspase-4 positive patients. Palmitic acid is a saturated long-chain fatty acid with a 16-carbon backbone and is the first fatty acid produced during lipogenesis (fatty acid biosynthesis). Malonic acid is the precursor of malonyl-CoA, which is important for fatty acid biosynthesis and elongation. AcetylCoA, an enzyme that is involved in the tricarboxylic acid (TCA) or Krebs’ cycle, is converted by carboxylation into malonyl-CoA by means of the acetyl-CoA carboxylase. Therefore, it looks like that according to the levels of the palmitic acid and of the malonic acid, rather than the malic acid, which is an intermediate of the TCA obtained from the conversion of fumarate by fumarase, the metabolic equilibrium in tissue-caspase-4 positive NSCLC patients is shifted towards a lipidomic profile (Figure 7). These data could be interpreted in two different manners. On one side the increase of the palmitic acid, and not of other fatty acids (i.e., stearic, linoleic and arachidonic acids), could be at the basis of caspase-4 activity. In particular, Zanoni et al. and Chu et al., reported that caspase-11, the murine analogue of caspase-4, may directly bind to 1-palmitoyl-2-arachidonoyl-sn-glycero-3-phosphorylcholine (PAPC) [16, 17], a phospholipid containing palmitic acid and arachidonic acid at the *sn*-1 and *sn*-2 positions [17], respectively, that is located in cell membranes and lipoproteins. It has to be pointed out though that the two above papers are contradictory. While Zanoni et al. [16] reported that oxPAPC binds to caspase-4 in dendritic cells with an ensuing activation of the enzyme, Chu et al. [17] assessed that the ligation of oxPAPC to caspase-11, the murine analogue of caspase-4, inhibited its activation in that to protect against septic shock. Instead, in our experimental conditions, caspase-4 positive tissues presented activated fragments of the enzyme (submitted paper) [8], ruling out its biological inhibition. In support, Pillon et al. [18] proved that obese individuals presented elevated levels of palmitate-induced caspase-4 activity in monocytes which undergo pyroptosis after IL-1β and IL-18 release. Instead, our data is referred to tumor tissues in which caspase-4 is associated to higher content of palmitic and malonic acid, but not associated to cell death, rather to tumor cell proliferation. Therefore, we could speculate that the increase in both malonic acid, as a precursor of malonil-CoA which is a key enzyme for lipogenesis, and of palmitic acid could be due to the ligation to caspase-4 (Figure 7), which comprises a CARD domain to which lipidic acids can bind [15]. Although it was reported that the CARD domain of caspase-4 in humans and caspase-11 in mice directly binds to cytosolic lipopolysaccharide (LPS) [19], so far, endogenous ligand/s for caspase-4 have not been identified; therefore, it is quite speculative to indicate the palmitic acid as a potential endogenous ligand for caspase-4. Nonetheless, in support, other fatty acids (i.e., stearic, linoleic and arachidonic acids) evaluated in this study were reduced in caspase-4 positive tumor tissues. To date, the stearic, linoleic and arachidonic acids are precursors of differential pro-inflammatory mediators such as prostaglandins, leukotrienes and lipoxins, highly detected in lung cancer tissues [11, 20–23].

On the other side, because of the shift towards fatty acid biosynthesis, it would be obvious to ask how tumor cell survive and where they can obtain cell energy. In this study, we found that the TCA cycle was supported by higher expression of SDHA in caspase-4 positive tumor tissues. In addition, we found that valine and proline, two essential aminoacids, were increased in tumor tissues, implying that the TCA cycle was supported by the glucogenic SDHA and valine/proline increase. In particular, valine and proline are glucogenic aminoacids that could derive from proteolysis in favor of the unbalanced TCA cycle in caspase-4 positive tissues, in order to provide the essential ATP.

In conclusion, we found that caspase-4 tumor tissues were associated with higher consumption of glucose in favor of fatty acid biosynthesis. This shifted equilibrium was balanced by the anaerobic LDH activity and valine/proline/SDHA-induced TCA cycle in order to provide the dysregulated glucose metabolism to provide ATP to the tumor cell in order to survive and proliferate. These data could be matched in a diagnostic tool in order to clinically discriminate caspase-4 positive NSCLC patients according to the lipidomic profile to open new therapeutic perspectives for NSCLC patients.

## MATERIALS AND METHODS

### Human samples

Patients in this study were diagnosed of resectable NSCLC at Ospedale dei Colli, AORN, Monaldi, Naples, Italy, during the period 2014–2017. Clinical data were obtained from questionnaires and histology reports from the Pathological Anatomy Unit of the hospital. The project was approved by the institutional review board and by the Ethical Committee (approval number for lung cancer patients 1254/2014). Blood from healthy non-smokers were obtained by the same Unit at Ospedale dei Colli, AORN, Monaldi, Naples, Italy, when possible, and by “Casa di Cura La Quiete”, Salerno, Italy, in collaboration (approval, 19.12.2016) with ImmunePharma s. r. l. Blood from healthy subjects and lung cancer patients was collected after oral and written information provided by the MDs, and signed a written consent form before entering the project. EDTA-blood was obtained prior to surgery, in case of lung cancer patients.

Healthy subjects were 40 ± 10 (mean ± S. E. M) years of age; NSCLC cancer patients were 60 ± 10 (mean ± S. E. M) years of age. Blood was withdrawn, collected and used within 24 hours.

### Metabolomic analysis

Metabolome extraction, purification and derivatization were carried out with the MetaboPrep GC kit (Theoreo srl, Montecorvino Pugliano, Salerno, Italy) according to the manufacturer’s instructions. Details regarding metabolite extraction and the overall analytical scheme, including QA/QC sample analyses, were reported in Troisi et al. [9]. Metabolomic profiles have been obtained by using gas chromatography coupled to mass spectrometry (GC-MS analysis) [24]. In brief, 2 μL of samples of the derivatized solution were injected into the GC-MS system (GC-2010 Plus gas chromatograph coupled to a 2010 Plus single quadrupole mass spectrometer; Shimadzu Corp., Kyoto, Japan). Chromatographic separation was achieved with a 30 m × 0.25 mm CP-Sil 8 CB fused silica capillary GC column with 1.00 μm film thickness (Agilent, J&W Scientific, Folsom, CA, USA). High-purity helium was used as carrier gas. The initial oven temperature of 100°C was maintained for 1 min and then raised by 4°C/min to 320°C with a further 4 min of hold time. The gas flow was set to obtain a constant linear velocity of 39 cm/s and the split flow was set at 1:5. The mass spectrometer was operated with electron impact ionization (70 eV) in full scan mode in the interval of 35–600 m/z with a scan velocity of 3333 amu/sec and a solvent cut time of 4.5 min. The complete GC program duration was 60 min. Untargeted metabolites were identified by comparing the mass spectrum of each chromatographic peak with the NIST library collection (NIST, Gaithersburg, MD, USA). Over 250 signals were observed in each sample, but several were not investigated further because they were either not consistently found in other sets of samples or were too low in concentration to be confirmed as metabolites due to poor spectral quality. A total of 243 endogenous metabolites involved in energy metabolism, lipid metabolism and amino acid metabolism were consistently detected and positively identified. To identify peaks, the linear index difference max tolerance was set at 10, while the minimum matching for the NIST library search was set at 85%.

### Proteomic analysis

Lungs were digested with 1 U/mL of collagenase (Sigma Aldrich, Rome, Italy). Lung homogenates underwent SDS-page. Coomassie-stained gels were used to isolate specific proteic bands (17-20-25-35-48 kDa). Trypsin digested bands were subjected to LC/MS/MS (LTQ ORBitrap XL; Thermo Fisher Scientific, MA, USA) and then analyzed by means of MASCOT. Protein identification and quantification were performed by matching mass spectrometry data in on-line protein database, using the MASCOT 1.5.2.8 software.

### LDH assay

LDH levels were evaluated in human lung homogenates and plasma by using commercially available LDH assay kit (Dojindo Molecular Technologies, Inc.). Briefly, samples were incubated with working solution for 15 minutes at room temperature, protected from the light. After stop solution addition, the formation of formazan, colorimetric derivative of LDH activity, was measured at the absorbance of 405 nm.

### Caspase-4 ELISA

The levels of plasma and tissue caspase-4 were evaluated by means of ELISA (patented by ImmunePharma S. r. l.; RM2014A000080 and PCT/IB2015/051262). The ELISA kit was based on custom antibodies, projected by the ImmunePharma S. r. l. and that are not currently commercially available.

### Western blotting

The expression of succinate dehydrogenase, subunit A (SDHA) (Santa Cruz Biotechnologies, Inc., Dallas, TX, USA) was evaluated in non-cancerous and cancerous lung homogenates.

### Statistical analysis

Results are expressed as median ± interquartile range. One Way ANOVA, followed by Bonferroni’s post-test, and/or Mann Whitney test were used where appropriate. *p* values less than 0.05 were considered significant.

## SUPPLEMENTARY MATERIALS


