# Supplementary material for: Altered lung tissue lipidomic profile in caspase-4 positive non-small cell lung cancer (NSCLC) patients
Source: Oncotarget. 2020 Sep 22;11(38):3515–25. doi: 10.18632/oncotarget.27724 (PMC7517963; doi:10.18632/oncotarget.27724)
Supplement: Supplementary file 1 [file oncotarget-11-3515-s001.pdf]

Altered lung tissue lipidomic profile in caspase-4 positive non-small cell lung cancer (NSCLC) patients

SUPPLEMENTARY MATERIALS

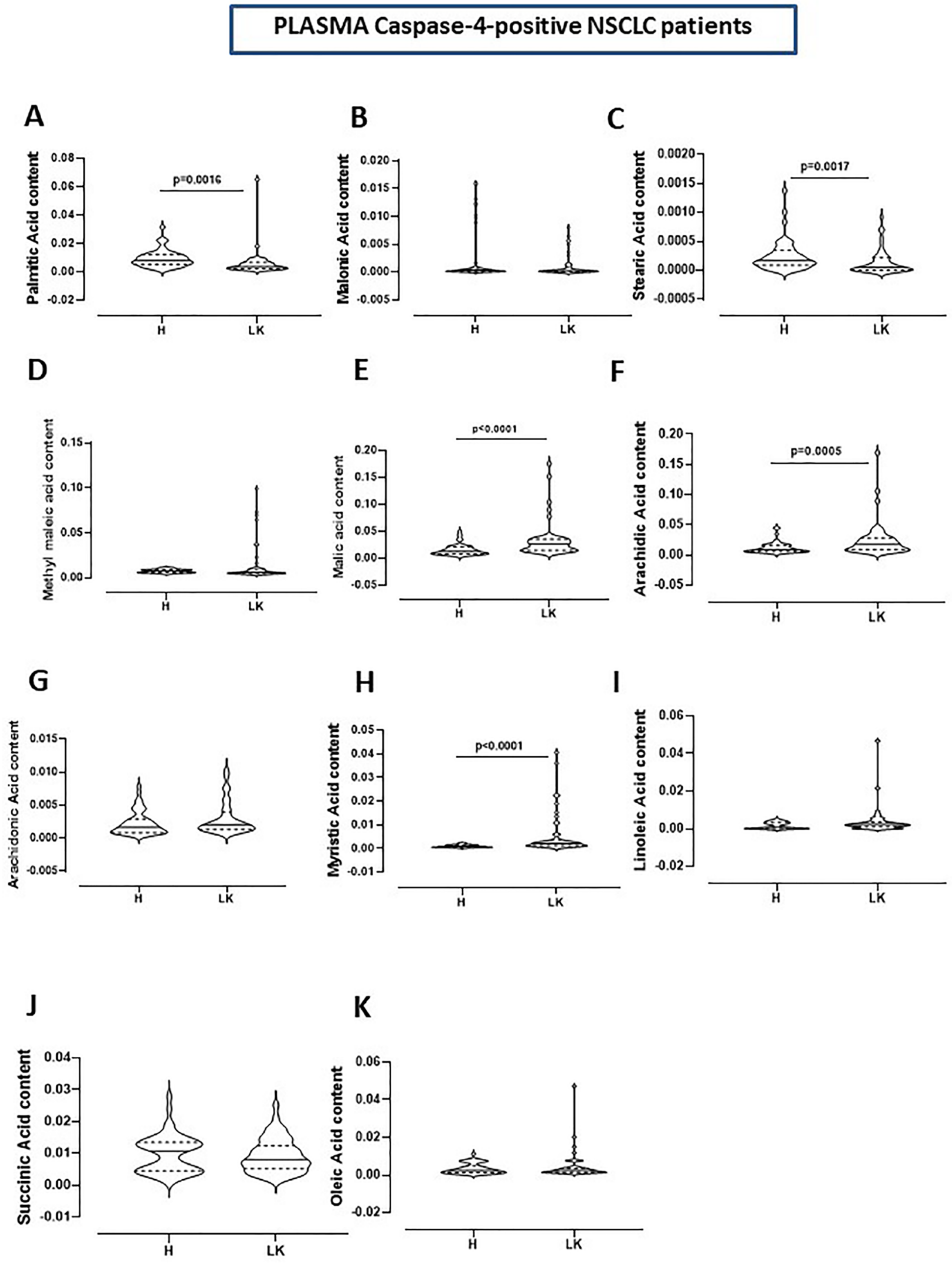

Supplementary Figure 1: Plasma levels of (**A**) palmitic acid, (**B**) malonic acid, (**C**) stearic acid, (**D**) methyl maleic acid, (**E**) malic acid, (**F**) arachidic acid, (**G**) arachidonic acid, (**H**) myristic acid, (**I**) linoleic acid, (**J**) succinic acid and (**K**) oleic acid detected by means of GC-MS. Data are showed as median ± interquartile range and represented as violin plots ($n = 50$). Two-tailed Mann Whitney U test was performed. $p < 0.05$ was considered as significant.
